# Hellenic National Observatory for Internet and Gaming Addiction

**DOI:** 10.1192/j.eurpsy.2025.948

**Published:** 2025-08-26

**Authors:** G. Floros, K. Siomos, N. Christodoulou

**Affiliations:** 1 2nd Department of Psychiatry, Medical School, Aristotle University of Thessaloniki, Thessaloniki; 2 Department of Psychiatry, Medical School, University of Thessaly, Larisa, Greece

## Abstract

**Introduction:**

The Greek National Observatory for Internet and Gaming Addiction (NOIAD) is a state-sponsored organization that will be setup in the University of Thessaly during this year following its inclusion in the Greek National Health Plan for Mental Health 2021-2030.

**Objectives:**

In this presentation of the setup and goals of NOIAD, a case will be brought forward for scientific collaboration across the European continent that could culminate in similar centers in different countries.

**Methods:**

The main publication that NOIAD intends to produce annually will have the role of a national report or at least an update of the national situation. In addition to this report, the observatory is expected to prepare a number of studies or reports on individual problems or in response to questions from state institutions (eg Parliament, ministries). Implementation of an online platform (portal) of specialized but also popular knowledge that will aim to inform health professionals and the general population (parents, pupils/students, adults with an addiction problem) with different information profiles for each population. Information lectures, educational activities for minor students, organization of an annual interdisciplinary conference.

**Results:**

NOIAD’s principal goal is the planning and implementation of actions to address digital addictions through the collection of objective, reliable and valid information about the state of Internet and Gaming addiction prevalence and related research and clinical practice in Greece. NOIAD will provide a state-of-the-nation annual report and disseminate the relevant information and conclusions to health professionals, government bodies and the wider population. Furthermore, NOIAD will plan and provide local preventative actions in the wider area of Thessaly, especially regarding the high-school and university student population; these actions will serve as blueprints for similar activities across Greece.

**Conclusions:**

Setting up a national center to provide with a complete picture of Internet Addiction and Gaming Disorder, pool together research output and coordinate preventative actions will assist with a fragmented research landscape and make better use of limited resources.

**Disclosure of Interest:**

None Declared

